# Observations on Necrosis

**Published:** 1786

**Authors:** M. Bousselin

**Affiliations:** Surgeon Major of the Polish Army, &c.


V. *Obſervations on Necroſis. By M.* Bouſſelin, *M. D. Surgeon Major of the Poliſh Army*, &c. —Vide *Memoires de la Société Royale de Medecine*, Vol. IV. 4to. Paris, 1785.

WE now know, that, in the long bones of the human body, a portion of the bony cylinder may be deprived of the living principle; and that, in ſuch caſes, nature gradually ſeparates the living from the dead part. While this ſeparation is taking place, a new cylinder is formed by an effuſion of bony matter, ſo as to incloſe the old bone, as it were, in a caſe; and the new bone connecting itſelf with the extremities of the old, the limb retains its original ſhape, and, after a certain time, when the bone has acquired the neceſſary degree of ſolidity, becomes as firm, and as capable of motion, as before.—The experiments by M. Troja, of which we formerly gave ſome account *, prove that this regeneration of the long bones may be produced by deſtroying their marrow.

Inſtances of this diſeaſe are to be found in the writings of Ruyſch, Scultetus, and others †; and

* Vol. III. page 357.

† M. Bouſſelin refers for caſes of this ſort to the *Mem. de l'Academie de Chirurgie*, Vol. V.; to a Latin Diſſertation *de Necroſi*, and a very curious cafe of it is defcribed and accurately delineated by our countryman, Mr. Chefelden *, who feems to have been aware of the utility of extracting the detached portion of dead bone, in order to relieve the patient in this difeafe. But it feems to have been only within thefe few years that furgeons have ventured to affift nature in thefe cafes, by making an opening in the new bone large enough to extract the loofe portion of the old.—In a former volume

*Necrofi*, by M. Chopart; and to the firft volume of the Edinburgh Medical Effays: but the cafe mentioned in the laft of thefe works, of a part of the tibia taken out, and afterwards fupplied by callus, cannot properly be quoted as an inftance of the difeafe in queftion; for in that cafe no new bone appears to have been formed till after the old one was removed.

* In his *Ofteographia*, chap. vii., where, after fpeaking of a cafe, "in which all the internal hard part of a cylindrical "bone having been feparated from the reft, and drawn out "through the place where the external caries made a vent, "the patient received a perfect cure," he obferves, that, "in another cafe of this kind, (delineated in table lv.) "where the internal part which contains the medulla was "alfo feparated from the reft, and there were holes through "which the matter was difcharged, but none fufficient to take "out the exfoliated bone; the matter continued to flow in "great quantity till it deftroyed the patient." "And poffibly," adds Mr. Chefelden, "if this cafe had been rightly known, "the volume * we gave an account of ſuch an operation, as deſcribed by the late M. David.

The paper now before us contains eight caſes of Necroſis, which have fallen under the author's obſervation; together with ſome judicious remarks on the nature and treatment of the diſeaſe.

From the circumſtance of his having ſeen twelve inſtances of it during a reſidence of two years in the Hotel Dieu at Lyons, M. Bouſſelin ſuppoſes it to be a diſeaſe of more frequent occurrence than has hitherto been imagined. Ten of theſe twelve patients were between thirteen and twenty years of age; and in eight of the twelve either the tibia or the os femoris was affected. Theſe obſervations lead him to imagine that young perſons are more liable to the diſeaſe than old, and that the lower extremities are more frequently affected by it than the upper; but ſuch concluſions are hardly allowable from ſo ſmall a number of facts.

The firſt caſe related by M. Bouſſelin is that of a young man aged eighteen years, who was

" the internal exfoliated part might have been taken out, and " the patient cured." — Theſe obſervations have eſcaped the notice of M. Bouſſelin.

* Vol. III. page 369.

 admitadmitted into the hofpital in April, 1781, with feveral ulcers in one of his legs, which was fwelled throughout the whole length of the tibia.

When the efchars floughed off, a fiftulous opening was difcovered towards the upper part of the tibia, through which a probe could be introduced into the cavity of the bone.

The difeafe, it feems, had begun about a year and a half before, by a very acute pain of the leg, followed by a fwelling of the foft parts, and foon after by external inflammation, which terminated in feveral fmall abfceffes, the difcharge from which removed in a great meafure the fwelling. Some of thefe ulcers, after difcharging a whitifh pus, and occafionally minute portions of bone, for five or fix months, healed, and were fucceeded by fimilar abfceffes in different parts of the leg.

In the beginning of the difeafe the limb was very weak, and incapable of fupporting the weight of the body; but, by degrees, it acquired more ftrength, and the fwelling of the leg gradually difappeared, except along the courfe of the tibia: the patient, however, we are told, was for four months unable to walk on this leg, but after that time gradually recovered vered the uſe of it, and, when he was admitted into the hoſpital, could walk on it almoſt as well as on the other leg.

The operation was performed on it on the 5th of May, by M. Bouchet, principal ſurgeon of the hoſpital. An oval inciſion was firſt made through the integuments, three inches and a half in length, and one and a half in breadth, which included ſeveral ſmall ulcers at the upper and fore part of the tibia. When this portion of the integuments was removed, the rugine was applied to the bone; after which M. Bouchet, with a ſmall convex ſaw, penetrated into the bone to the depth of half an inch, both at the upper and lower parts of the wound, and then with a mallet and chiſſel removed the intermediate ſpace of bone, which was accompliſhed with great difficulty, on account of its uncommon hardneſs.

On removing this piece of bone, the ſurgeon found that he had not yet got into the cavity; the operation was therefore extended to a greater depth, and then a looſe portion of bone was removed, three quarters of an inch long, and four tenths of an inch broad, but rendered extremely thin by ſuppuration.

Common dreſſings were applied to the wound, and ſuffered to remain till they were ſufficiently moiſt to be removed with eaſe, which did not happen till the fifteenth day after the operation.

About the third day the patient began to complain of a ſlight ſymptomatic fever, but this ſoon yielded to an antiphlogiſtic regimen.

At the end of ſix months, when the wound was nearly healed, the patient quitted the hoſpital.

The ſubject of the ſecond caſe was a girl, thirteen years old, and ſeemingly of a good habit of body, but who, for the ſpace of a year, had ſeveral ulcers on her left leg, with fiſtulous ſinuſes on the tibia, which was conſiderably enlarged. Small exfoliations of bone had made their way through ſome of the ulcers, which afterwards healed, as in the preceding caſe, and were followed by freſh ulcers in other parts of the leg. No operation was attempted in this caſe, as two of the ulcers healed during the two months ſhe remained in the hoſpital, and ſhe recovered the uſe of her limb ſo much, that it was thought ſhe would get well in time.

The third cafe is that of a young man, aged eighteen years, who, at the time he was admitted into the hofpital, laboured under a hectic fever, and had three or four ulcers in each leg. Both tibias were enlarged, and a probe, introduced at the fiftulous openings, paffed into the bone. The fores difcharged a laudable pus, but the ulcers were filled with fungus.

The patient dated the origin of his complaint from a fever, with which he had been attacked about three years before, and which had been followed by abfceffes in both his legs. The difcharge from thefe fores was very profufe, and occafionally minute portions of bone came through the wounds. Some of the ulcers gradually healed, and frefh ones appeared in other parts of his legs. While the abfceffes were forming, he complained of acute pain; but when they came to difcharge and ulcerate, they occafioned but little uneafinefs. His legs, from being extremely weak, had gradually acquired ftrength, and, during the three months he remained in the hofpital, feveral of the ulcers healed, and he went out at the end of that time much relieved.

The fubject of the fourth cafe was a man, thirty-fix years old, who was admitted into the hofpital hoſpital with an ulcer, of ſix months ſtanding, on the inner and lower part of the thigh. The whole of the limb was enlarged, and in the center of the ulcer was a fiſtulous ſinus leading to a cavity, in which a moveable bone might be felt. — The bone was laid bare, and a ſuffi-cient portion of it removed to enable the ſur-geon to take out the moveable piece of bone, which was found to be part of the cylinder of the os femoris, an inch long, but ſomewhat thinner than in its natural ſtate, part of its ſubſtance having been deſtroyed by the ſup-puration.

The wound in this caſe was healed in about three months, and the patient recovered the perfect uſe of his limb.

The fifth caſe is that of a ſtout man, aged twenty-ſix years, who was admitted into the hoſpital with a compound fracture of one of his legs. At the end of three months he was diſcharged from the hoſpital, though he had ſtill an ulcer on the leg. He returned again in about a month, and the ulcer was then found to have a fiſtulous ſinus leading to a moveable piece of bone, and the tibia, at the place where it had been fractured, was obſerved to be conſi-derably enlarged.

The neceſſary inciſions were made to bring to view the moveable piece, which was then found to be ſurrounded by a portion of regenerated bone. The new bone, not having acquired much hardneſs, an opening was eaſily made in it large enough to extract the looſe piece, which proved to be a portion of the tibia, an inch long, and half an inch broad. The cure in this caſe was effected in about ten weeks.

The ſixth patient, whoſe caſe is related by our author, was a young man, aged fifteen years, who was brought to the hoſpital with both his legs covered with ulcers, and unable to walk. The diſorder, he ſaid, had come on about two months before with acute pain, which continued till ſuppuration took place and matter was diſcharged.

In each leg there were five or ſix fiſtulous openings, leading to the cavity of the tibia, in which were moveable pieces of bone. The tibiæ were but ſlightly enlarged.

It was agreed in this caſe that an operation ſhould be performed on one leg firſt; and accordingly, about a month after his admiſſion, a circular inciſion, three inches long, and one inch and a half wide, was made through the integuments. On removing this flap, ſeveral openings openings were difcovered in the regenerated bone, through which the loofe portion of bone might be feen. All thefe openings were laid into one, by removing, with a chiffel and mallet, the fpace between them, which was done with great facility on account of the foftnefs of the new bone. The loofe piece, which was taken out, preferved the fhape of the old bone, and was three inches long, but had loft part of its fubftance by fuppuration.

A flight fymptomatic fever fucceeded the operation, and continued during ten or twelve days; after which it fubfided. The wound healed in about fix months.

The ulcers in the other leg ftill continued to difcharge a good deal of matter, and occafionally fmall portions of bone. Some of the ulcers were healed, and the limb had acquired ftrength; but the fuccefs of the operation on the other leg made the patient defirous to undergo a fimilar operation in this leg alfo. It was accordingly undertaken, and a flap, including all the remaining ulcers, removed; but no opening was found leading to the cavity of the bone, excepting a very fmall one at the upper part of the wound. A portion of the new bone, however, was removed, but not without difficulty, as as it had acquired confiderable hardnefs, and fome fmall pieces of loofe, dead bone were found, almoft wholly diffolved by the pus; fo that nature had here nearly effected a cure.

The next cafe is that of a boy, thirteen years old, who was fuddenly feized with acute pain in both legs, to which fucceeded inflammation and fuppuration. When he came into the hofpital, in May, 1781, the diforder was of three months ftanding, and he had feveral fiftulous finufes in each leg, which led to cavity, in which a moveable piece of bone might be felt with a probe.—His legs were not fwelled; but the fkin at the fore part of each tibia was extremely thin, and here and there were fpots which afforded lefs refiftance than the other parts of the bone, and where the offification feemed to be deficient. The pus difcharged from the finufes was whitifh, and of a good confiftence.

In this cafe an oval incifion, fix inches long, and which included all the finufes, was made through the integuments; and when this flap was removed, the furgeon found the new bone had acquired fo little folidity, that he was able to cut through the anterior portion of it with a common biftoury.—The periofteum was thickened and oſſified in ſeveral places. After the removal of a looſe portion of dead bone, the wound was dreſſed with dry lint, which came off on the ſixth day. The bottom of the wound was ſoon filled with fleſhy granulations, which covered the cavity of the new bone.

In the courſe of the treatment, the wound was twice in a putrid ſtate, which the author aſcribes to the bad air of the hoſpital, and to ſome irregularities of diet; but by proper care the cure was completed in about ſix months; ſo that at the beginning of November the patient was able to walk without a crutch, and the limb, we are told, is not deformed.

During the whole of this treatment care was taken to keep open the ſinuſes of the other leg, the diſcharge from which was a good pus, accompanied from time to time with portions of bone, which, in general, were very ſmall; but one piece that made its way to the mouth of one of the ſinuſes, and was extracted with the forceps, was nearly two inches in length.

The operation was performed on the other leg November 15th. The new bone was here found to have acquired ſo much ſtrength, that the mallet and chiſſel were required to make an opening into the cavity of the bone, but no looſe loofe piece of bone was found in it; fo that nature had in this inftance been able to rid herfelf of the whole of the detached portion. The cure went on in a favourable manner, and when the account of this cafe was drawn up, the wound was nearly healed.

The eighth and laft cafe mentioned by M. Bouffelin is that of a young man, eighteen years old, who hurt his knee by a fall, which occafioned great pain, and laid the foundation of an abfcefs in the lower and inner part of the thigh. The difcharge from this abfcefs was very profufe for feveral months; he had a ftiff joint, and there remained a fiftulous finus, and an enlargement of the condyles of the tibia and femur.

In this ftate he was admitted into the hofpital in Auguft, 1781; at which time a moveable piece of bone could be felt on introducing a probe through the finus, and left no room to doubt of the nature of the complaint. The operation was performed the month following, and a portion of detached bone, between three and four inches in length, and which was found to be the lower extremity of the os femoris, was extracted. The patient left the hofpital about the end of November;

 at at which time the wound was nearly healed, but the motion of the joint was entirely loft.

After defcribing thefe inftances of the difeafe, M. Bouffelin gives us fome obfervations on the diagnofis and prognofis in cafes of this kind, and on the method of cure.

The principal characteriftic marks of the difeafe, which our author points out, are, 1. the fituation of the fiftulous ulcers which ufually occupy that part of the bone neareft to the integuments, and penetrate into its cavity, where the detached portion of bone may in general be felt; 2. the difcharge, in the greater number of cafes, of a good pus, the quantity of which is not increafed by compreffion, and which does not give a black tinge to the dreffings, as in caries, unlefs, as feldom happens, we are told, the difeafe is complicated with caries; 3. the occafional difcharge of fmall portions of bone that make their way through the finufes; 4. the feat of the pain; and laftly, the circumftance of the tumefaction's being confined to the bone, and not extending to the foft parts.

The enlargement of the bone, it is obferved, ufually correfponds with the extent of the difeafe; but this enlargement is not perceptible till the new bone has acquired a certain degree of hardnefs.

On the fubject of the prognofis M. Bouffelin obferves, that if the difeafe is of long ftanding, if the fuppuration is much diminifhed, if portions of bone have made their way through the finufes, and the new bone has acquired great firmnefs, and is larger than natural, there will be reafon to conclude that the detached bone is diffolved, or nearly fo, and that nature will be able to complete the cure. If an operation were to be undertaken, under fuch circumftances, not only no loofe bone would be found, as happened in the fecond operation performed on the fubject of the feventh cafe, but the furgeon would find it difficult to get into the cavity of the bone, as this becomes obliterated in proportion as the portion of dead bone is leffened by the fuppuration.

In general the cure by nature will be more or lefs difficult, we are told, in proportion as the difeafe is more or lefs extenfive. It will likewife, our author thinks, be, *cæteris paribus*, more difficult in adults than in younger fubjects. As a proof of this he refers to the fixth cafe, where, in a man of thirty-one years, the detached portion of bone was hardly at all diminifhed minifhed in fix months, while in the eighth cafe, the fubject of which was only fifteen years old, the bone, in the fame fpace of time, was almoft wholly deftroyed, and yet the difeafe in both cafes was nearly of the fame extent.

With refpect to the mode of treatment, our author remarks, that the writers on this fubject have hitherto thought only of the operation; but he is convinced that nature will oftentimes be equal to the cure, though by flower means. In fome cafes, he obferves, fix or eight months will be fufficient for her purpofe; in others fhe may require one or two years. He acknowledges, however, that cafes may occur in which the long duration of the difeafe, the degree of fuppuration, and other circumftances, may render the operation neceffary.

In cafes where the difeafe is very extenfive, and in which a great lofs of fubftance muft neceffarily take place in the operation, he prefers the leaving the difeafe to nature, efpecially if the patient be young; but in older perfons he gives the preference to the operation, efpecially if it be undertaken at an early period of the difeafe, becaufe the new bone being then not fufficiently formed to inclofe every part of the old, the loose piece may be eafily extracted, and the new bone's want of firmnefs will allow it to be eafily cut into, if the removal of any part of it fhould be found neceffary.

M. Bouffelin cautions us, however, not to undertake the operation till we are fure that nature has thrown off the whole of the dead portion of bone. This, he obferves, may be known by the good quality of the pus, by the difappearance of inflammation on the furface of the adjacent fkin, and by the firmnefs of the limb. This laft he confiders as the principal fign of the feparation being complete.

The author does not attempt to defcribe the manner of operating in thefe cafes; becaufe this, he obferves, muft vary according to circumftances. He thinks it fufficient to remark, that the aim of the operator fhould be to expofe fufficiently the difeafed part, by cutting into or removing the foft parts, and to make the opening in the bone extenfive enough to include all the finufes in its fubftance. This opening, he adds, muft alfo be proportioned to the fize of the loofe portion of bone,

